# Restrictive Cardiomyopathies: The Importance of Noninvasive Cardiac Imaging Modalities in Diagnosis and Treatment—A Systematic Review

**DOI:** 10.1155/2017/2874902

**Published:** 2017-11-15

**Authors:** Aidonis Rammos, Vasileios Meladinis, Georgios Vovas, Dimitrios Patsouras

**Affiliations:** ^1^Department of Cardiology, Chatzikosta General Hospital, Ioannina, Greece; ^2^Department of Cardiology, Agrinio General Hospital, Agrinio, Greece

## Abstract

Restrictive cardiomyopathy (RCM) is the least common among cardiomyopathies. It can be idiopathic, familial, or secondary to systematic disorders. Marked increase in left and/or right ventricular filling pressures causes symptoms and signs of congestive heart failure. Electrocardiographic findings are nonspecific and include atrioventricular conduction and QRS complex abnormalities and supraventricular and ventricular arrhythmias. Echocardiography and cardiac magnetic resonance (CMR) play a major role in diagnosis. Echocardiography reveals normal or hypertrophied ventricles, preserved systolic function, marked biatrial enlargement, and impaired diastolic function, often with restrictive filling pattern. CMR offering a higher spatial resolution than echocardiography can provide detailed information about anatomic structures, perfusion, ventricular function, and tissue characterization. CMR with late gadolinium enhancement (LGE) and novel approaches (myocardial mapping) can direct the diagnosis to specific subtypes of RCM, depending on the pattern of scar formation. When noninvasive studies have failed, endomyocardial biopsy is required. Differentiation between RCM and constrictive pericarditis (CP), nowadays by echocardiography, is important since both present as heart failure with normal-sized ventricles and preserved ejection fraction but CP can be treated by means of anti-inflammatory and surgical treatment, while the treatment options of RCM are dictated by the underlying condition. Prognosis is generally poor despite optimal medical treatment.

## 1. Introduction

According to a recent position statement of the European Society of Cardiology (ESC) working group on myocardial and pericardial diseases, cardiomyopathy is defined as a myocardial disorder in which there is abnormality in the structure and function of the myocytes, in the absence of coronary artery disease, hypertension, valvular disease, and congenital heart disease that could justify this abnormality. In the past, cardiomyopathies were also separated from systematic disorders that could involve the myocardium. ESC classification proposes a shift from diagnosing by exclusion criteria and focusing on the morphology and function of the heart [1].

Restrictive cardiomyopathy (RCM) is the least common of cardiomyopathies. It is characterized by impaired diastolic function with restrictive filling and reduced diastolic volume of either or both ventricles, preserved systolic function, and invariably normal or mildly increased wall thickness [2]. Due to increased myocardial stiffness, a small increase in volume leads to a precipitous rise of pressure within the ventricle with an accentuated filling in early diastole, which ceases abruptly at the end of the rapid filling phase showing the characteristic “dip-and-plateau” pattern [3] during cardiac catheterization.

There are many classification criteria for the causes of RCM. According to the ESC Working group in myocardial and pericardial disease RCM can be idiopathic, familial (autosomal dominant, autosomal recessive or X-linked inheritance), or secondary to systematic disorders [1] (Table 1). For the purposes of this review, we will refer to the most common of them.

## 2. Clinical Presentation and Physical Examination

Patients commonly present with dyspnea, fatigue and limited exercise capacity, palpitations, and syncope, while angina may also occur. Others may present with thromboembolic complications. Physical examination may reveal signs of congestive heart failure (distended jugular veins occasionally with Kussmaul sign, peripheral bilateral edema, respiratory rales, hepatomegaly, ascites, and S_3_ and S_4_ gallops). The ECG may reveal sinus rhythm with signs of left or biatrial enlargement or atrial fibrillation. There may be nonspecific ST and T wave abnormalities. QRS voltage may be low in the precordial leads and conductive disturbances may also occur, particularly in infiltrative diseases. Specific ECG findings may occur depending on the underlying cause and aid in the diagnostic work-up.

## 3. Diagnosis

### 3.1. Laboratory Tests

A complete evaluation should be performed as restrictive cardiomyopathy may originate from systematic disorders or can in turn affect other organs as heart failure develops. Hematocrit (HCT), serum electrolytes, blood urea nitrogen (BUN), creatinine, 24 hr urine total protein, and liver function should be assessed. Arterial blood gas (ABG) should also be obtained to monitor hypoxia. Serum brain natriuretic peptide (BNP) or N-terminal pro-b-type natriuretic peptide (NT-proBNP) and troponin T are indicative of the heart failure and in many cases predictors of survival [5–7]. Specific disorders will require more sophisticated exams (angiotensin converting enzyme (ACE) in sarcoidosis [8], complete blood count (CBC) with peripheral smear helping to establish eosinophilia [9] in hypereosinophilic syndromes, serum iron concentrations, total iron-binding capacity and ferritin levels in hemocromatosis, immunoglobulin free light *κ*, *λ* chain testing, and serum and urine immunofixation in amyloidosis, etc.).

### 3.2. Echocardiography

**Figure 1:**
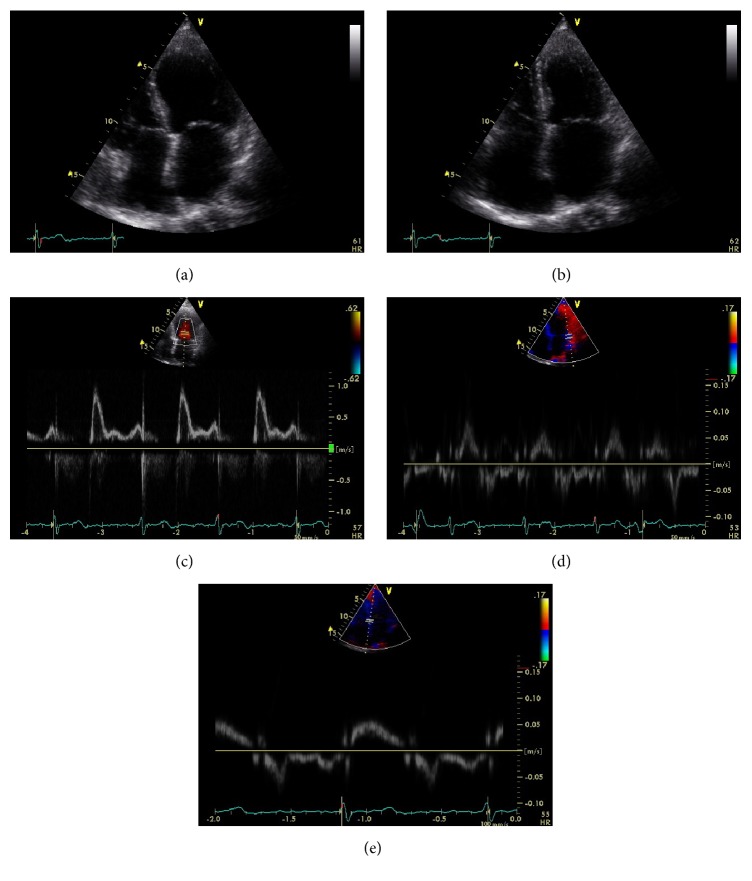
Echocardiographic images from a patient with restrictive cardiomyopathy. Two-dimensional 4-chamber view in diastole (a) and systole (b), showing normal left ventricular volume, wall thickness, and systolic function (EF 62%). There is marked biatrial enlargement. Pulse wave Doppler from the left ventricular inflow (c) showing restrictive filling pattern with an E wave velocity of 1 m/sec, an A wave velocity of 0.4 m/sec, and an E wave deceleration time of 145 msec. Spectral tissue Doppler from the lateral (d) and septal (e) mitral annulus. There is marked reduction in systolic annular velocities indicative of latent systolic dysfunction.* E*/*e*′ (*e*′ measured as the average between the two annular* e*′ velocities) is 16, indicative of increased left ventricular filling pressure. Note the marked reduction in lateral* a*′ velocity (<4 cm/sec) indicative of left atrial systolic dysfunction.

Echocardiography is the first imaging modality for the assessment of patients with dyspnea and/or heart failure. In patients with RCM, it usually discloses nonspecific findings such as normal (not dilated) ventricles with normal or increased wall thickness (Figures 1(a) and 1(b)) although it can occasionally give specific clues to the diagnosis. Transmitral spectral Doppler often shows restrictive filling pattern (accentuated early diastolic velocity with low or absent late filling velocity with E/A ratio > 2, E wave deceleration time < 150 ms, and short isovolumic relaxation time < 60 ms, Figure 1(c)) although this is commonly a sign of advanced myocardial involvement. There is marked left or biatrial dilatation usually as a consequence of chronically elevated filling pressures (Figures 1(a) and 1(b)). Although classically systolic function as expressed by means of ejection fraction (EF) is normal or near normal, novel techniques like tissue Doppler and speckle tracking echocardiography (STE) reveal the presence of latent systolic myocardial impairment which may be specific to the disease state. Tissue Doppler of the mitral annulus shows reduced systolic waves (s wave), blunted early diastolic waves, and preserved or blunted late diastolic waves, depending on the degree of atrial involvement in the myopathic process [10, 11] (Figures 1(d) and 1(e)).

One of the main utilities of echocardiography is the differential diagnosis between constrictive pericarditis (CP) and RCM. Both present as heart failure with normal-sized ventricles and preserved EF, dilated atria, and Doppler findings of increased filling pressure (often restrictive filling pattern). Differential diagnosis is important since CP can be treated by means of anti-inflammatory drugs or surgery while the treatment options of RCM are dictated by the underlying condition. For the main differences between those disease states, the reader can refer to Table 2 and recent research findings [12, 13].

### 3.3. Cardiac Magnetic Resonance

Echocardiography is unable to establish the definite diagnosis of specific RCM subtypes due to its inherent poor tissue characterization and limited assessment of the ventricular apex and the right ventricle. Cardiac Magnetic Resonance (CMR) with a higher spatial resolution and late gadolinium enhancement (LGE) can provide detailed information about anatomic structures, perfusion, ventricular function, and tissue characterization [14]. Late gadolinium enhancement (LGE) depending on the pattern of scar formation can direct the diagnosis to specific subtypes of RCM. Finally it can accurately measure pericardial thickness [15, 16] and visualize pericardial inflammation [17], aiding in the diagnosis of CP.

### 3.4. Cardiac CT

As of this writing, Cardiac CT has no specific role in the work-up of patients with RCM. It can accurately exclude the presence of coronary obstructive artery disease in patients with angina symptom. Its main role is in the differential diagnosis between RCM and CP where it can give invaluable information about the thickness and composition of the pericardium [18].

### 3.5. Invasive Catheterization

In contemporary practice, invasive catheterization and coronary angiography are rarely needed for the establishment of diagnosis. In cases where chest pain is the predominant symptom, it can reveal the presence or absence of obstructive coronary artery disease. Left and right heart catheterization can be used when findings of noninvasive tests, including the differential diagnosis between CP and RCM, are nonconclusive [19].

### 3.6. Cardiac Biopsy

Histological examination can be valuable in setting a definite diagnosis when noninvasive studies have failed, although there are periprocedural risks of endomyocardial biopsy (EMB) [20]. Moreover, in diseases where there is focal involvement of the myocardium (e.g., sarcoidosis), biopsy may miss the area of lesion, giving false negative findings.

## 4. Prognosis 

Prognosis varies among the specific types of RCM but is generally poor with progressive deterioration, particularly in children, despite optimal medical treatment.

## 5. Specific Types of Restrictive Cardiomyopathy

### 5.1. Cardiac Amyloidosis

This is the most common cause of RCM. It can be either systemic or localized to the heart muscle. It is characterized by deposition of amyloid fibrils in the extracellular space. These fibrils are culprit proteins misfold into *β*-pleated sheets antiparallel to one another, resistant to proteolysis, and they cause oxidative stress, disruption of the myocardium, and myocardial damage [21, 22]. There are many proteins that can form amyloid but not all of them affect the heart. The main difference among them is the origin of the protein formation, the percentage of heart involvement, and the rate of progress. Amyloid stains pink with hematoxylin and eosin and demonstrates apple-green birefringence when viewed under polarized light.

The deposition begins in the subendocardium and extends within the myocardium between the muscle fibres. Rarely, the pericardium may be the primary site of amyloid deposition without any endomyocardial involvement [23]. Increased wall thickness is therefore the result of interstitial deposition and not of hypertrophied myocardial cells, in contrast to hypertensive heart disease or hypertrophic cardiomyopathy (HCM). Small intramyocardial vessels may also be infiltrated leading to anginal symptoms while epicardial arteries are angiographically normal.

**Figure 2:**
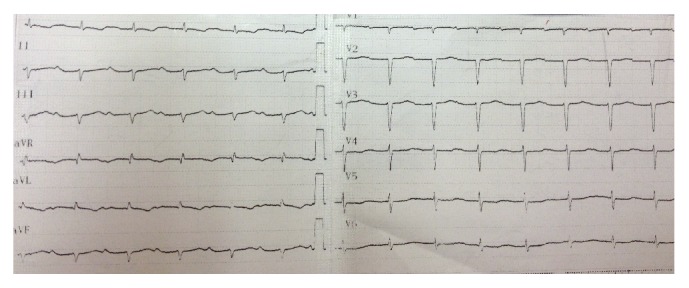
ECG from a patient with cardiac amyloidosis. There are low voltage QRS complexes with left axis deviation and marked 1st-degree atrioventricular block.

The conductive system is also affected causing several degrees of atrioventricular (AV) block. Other characteristic ECG abnormalities are low voltage QRS complexes which, in combination with the finding of increased wall thickness in echocardiography, can differentiate this disease from HCM. These findings are more commonly seen in light chain amyloid (AL) [24] than in familial transthyretin-related amyloidosis (ATTR) [25] subtype. Other ECG findings include a pseudoinfarct pattern (poor R wave progression in precordial leads), intraventricular conduction delay, and atrial arrhythmias while ventricular arrhythmias are rare [26–30] (Figure 2).

**Figure 3:**
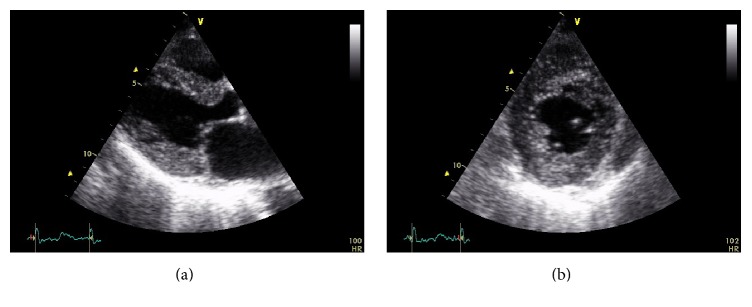
Parasternal long (a) and short axis (b) view from the patient whose ECG appears in Figure 2. There is marked LV hypertrophy. Also note the granular, speckled myocardial appearance.

Echocardiographic amyloidosis may present with biventricular hypertrophy, although up to 1/3 of cases may have normal wall thickness. The constellation of low voltage QRS complexes with hypertrophy, often with a restrictive filling pattern, is a classic feature but it indicates advanced disease. Left ventricular EF is commonly preserved in the early stages of the disease process as it is primary calculated from the contraction of the heart along its short axis, while the subendocardial myocytes that are prone to damage are longitudinally oriented and therefore the longitudinal contraction will be impaired [31]. The endocardium may also be infiltrated and there may be thrombi. A granular, speckled appearance of the ventricular myocardium, (Figure 3) while nonspecific (also seen in glycogen storage disease, HCM, Anderson-Fabry disease, hypertensive heart disease, and end-stage renal disease) [32], combined with clinical suspicion and other laboratory findings, is suggestive of cardiac amyloidosis.

Atrioventricular valves, if infiltrated, may be thickened with mild to moderate regurgitation. Mild or moderate pericardial effusion may be present. Thickening of the interatrial septum without sparing of the area of the fossa ovalis is characteristic of the disease but apparent only in late stages [33].

**Figure 4:**
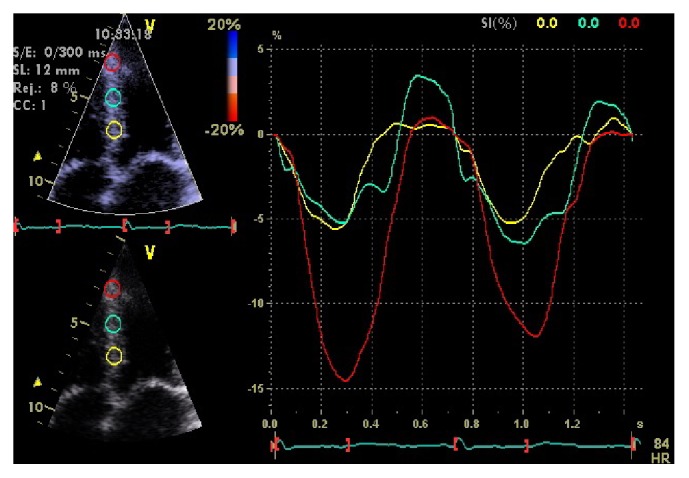
Strain curves from the interventricular septum of the same patient appearing in Figures 2 and 3. In this example, strain curves were constructed with the Doppler Myocardial Imaging technique. There is reduced peak systolic strain of the basal (yellow curve) and mid (green curve) septal segments, compared to the apical segment (red curve) with an apex to base ratio > 2.1.

Speckle tracking echocardiography, with the ability of measuring novel indices of deformation, has expanded its role in the diagnosis. It has been found that in cardiac amyloidosis systolic longitudinal strain (LSsys) is impaired in the mid and basal intraventricular septum compared to the apex (Figure 4). In a recent study, septal apical to basal LSsys ratio > 2.1 combined with deceleration time of early filling < 200 msec differentiated amyloidosis from other forms of LV hypertrophy with a sensitivity of 88%, specificity of 85%, positive predictive value of 67%, and negative predictive value of 96% [34] (Figure 4).

**Figure 5:**
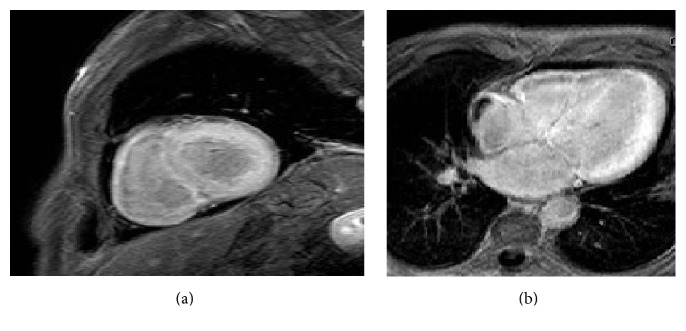
CMR short axis (a) and four-chamber view (b). LGE images showing diffuse, nonhomogenous myocardial enhancement involving both ventricles and atria. The pattern of enhancement is consistent with cardiac amyloidosis. Note the presence of a right atrial thrombus. Images courtesy of Professor Dr. Jan Bogaert, University Hospital Leuven.

CMR will show diffuse LGE throughout both ventricles, particularly the subendocardium with a characteristic zebra-stripe appearance of the subendocardial enhancement of the LV and RV endocardium, sparing the mid-wall of the interventricular septum [35]. Sometimes there is a patchy transmural pattern [36–38]. There is a faster contrast washout from myocardium and blood pool than normal. Enhancement of the atrial wall has also been reported [39] (Figure 5). The technique of T1 mapping may reveal cardiac involvement at an earlier stage compared to overt LGE images [40] and may also prove useful in patients with end-stage renal dysfunction where the administration of gadolinium based contrast is contraindicated due to the risk of nephrogenic systemic sclerosis. Recent studies have shown high sensitivity of the technique in the diagnosis of AL [41] and its ability to differentiate between AL and ATTR [42]. Moreover the combination of T1 mapping with LGE may have prognostic implications [43].

Radionuclide imaging using single-photon emission computed tomography (SPECT), or positron emission tomography (PET), has been recently introduced in the diagnostic work-up. An intense myocardial uptake of imaging agents Tc-99m pyrophosphate (PYP, available in the USA) or Tc-99m 3,3-diphosphono-1,2-propanodicarboxylic acid (DPD, available in Europe) identifies patients with ATTR and can differentiate them from those with AL [44, 45]. A recent study has shown that Tc-99m DPD uptake is an independent predictor of adverse events [46].

Histological confirmation is mandatory for the diagnosis. Renal, myocardial, or liver biopsy is invasive, expensive with high periprocedural risks [47, 48]. Preferably biopsy can be obtained by the iliac crest bone marrow [49] combined with subcutaneous abdominal fat aspiration [50] which will identify amyloidosis in 85% of cases [51]. If negative, biopsy directly from an involved organ should be obtained. A negative myocardial biopsy in suspected cases practically excludes cardiac amyloidosis, since myocardial involvement is usually widespread. In a positive examination though it is crucial to determine the type of amyloid as this in turn defines treatment and prognosis.

#### 5.1.1. Light Chain (AL) Amyloidosis

AL amyloidosis (formerly called primary) is the commonest and most aggressive form. Amyloid is derived from monoclonal light chains (or chain fragments) secondary to a plasma cell disorder (produced at the bone marrow). The most common plasma cell disorder is multiple myeloma; however only a minority of these patients will develop amyloidosis and vice versa. Cardiac deposition occurs in almost every case of biopsy or autopsy while clinical involvement is found in about half of the cases and has a major prognostic implication [30] with a median survival as low as 4 months [52].

The clinical presentation is that described generally for RCM above. Extracardiac organs infiltrated are the kidneys, skin, liver, peripheral, and autonomic nervous system and symptoms may rise, respectively.

Treatment must combine the underlying plasma dyscrasia and the subsequent heart failure, though patients with amyloidosis respond minimally to conventional heart failure treatment. There is no evidence of beneficial effect of beta blockers, except in atrial fibrillation for relative rate control; angiotensin converting enzyme (ACE) inhibitors and angiotensin receptor blockers (ARBs) are difficultly tolerated because of hypotension which is almost always present (in grounds of low cardiac output and autonomic neuropathy). Calcium channel blockers are contraindicated as they may produce a negative inotropic effect. Cautious titration to diuretics is the most effective treatment. Digoxin should only be used for rate control in atrial fibrillation with extreme caution since the risk of toxicity is high due to possible binding of the drug to amyloid fibrils. Melphalan plus dexamethasone supported with autologous stem-cell transplantation [31, 51] is considered a standard treatment which can increase the median survival. Novel agents such as thalidomide, lenalidomide, and bortezomib are also promising but most of the trials are still ongoing. Recently the results of a phase 1 trial involving the use of a drug called (R)-1-[6-[(R)-2-carboxy-pyrrolidin-1-yl]-6-oxo-hexanoyl]pyrrolidine-2-carboxylic acid (CPHPC) have been reported. The drug succeeded in triggering clearance of amyloid deposits in patients with systemic amyloidosis. Future trials will test its effectiveness in cardiac amyloidosis [53]. The ideal treatment would be a heart transplantation (though most of the patients are unsuitable by precise criteria suggested) with autologous stem-cell transplantation since the underlying systemic disorder still exists.

#### 5.1.2. Familial (ATTR) Amyloidosis

ATTR amyloidosis originates from a number of DNA mutations (autosomal dominant inheritance, more than 100 mutations) of the transthyretin (TTR) protein which is synthesized in the liver. The cardiac deposits vary depending on the mutation and the phenotype may be from exclusive neuropathy to cardiomyopathy or combination of the two [54]. Carpal tunnel syndrome may be an early indicator. Comparatively to AL amyloidosis ATTR has a more favorable prognosis of 3–5 years as it has a slower clinical course. Heart failure symptoms are treated with beta blockers and ACE inhibitors which are better tolerated than in AL amyloidosis. Liver transplantation is effective in reversing clinical disease in patients with TTR Val30Met mutation but it can accelerate progression of heart disease in the rest. Similar to AL amyloidosis the ideal treatment would be heart transplantation (neuropathy is a major contraindication) along with liver transplantation preferably at an early stage.

#### 5.1.3. Senile Systemic Amyloidosis (SSA)

SSA amyloidosis, formerly called wild type amyloidosis, is an exclusive cardiac disease with carpal tunnel syndrome being the only extracardiac manifestation. SSA has a more indolent course than AL with a median survival of 4–6 years after diagnosis [55] and is seen almost always in elderly males. Treatment is currently palliative, with diuretics being the cornerstone of therapy and as the clinical expression is mild, ACE inhibitors or ARBs, beta blockers, or nitrates are generally well tolerated. If atrial fibrillation occurs oral anticoagulants are indicated. Amiodarone is preferred over other antiarrhythmic agents to maintain sinus rhythm. If permanent pacemaker is needed because of high degree AV block, biventricular pacing should be considered as right ventricular pacing may worsen an already affected cardiac output.

Less common forms of amyloidosis are the AA (formerly called secondary) which is a result of chronic inflammatory conditions affecting mostly the liver and kidneys with less than 2% of the cases involving the heart and the isolated atrial amyloid where atrial natriuretic peptide (ANP) is synthesized by atrial myocytes and can be deposited as amyloid and is nowadays considered part of the ATTR or SSA subtypes.

### 5.2. Sarcoidosis

Sarcoidosis is a systematic noncaseating granulomatous disorder affecting several tissues. Most commonly the respiratory system is affected and patients typically present with hilar lymphadenopathy on chest X-ray. Skin, gastrointestinal, ocular, and nervous system involvement follows in frequency. Cardiac sarcoidosis (CS) is clinically present in about 5% of patients, although an autopsy study showed a prevalence of cardiac involvement in approximately 25% of cases [56] which portends worse prognosis [57].

Sarcoid granulomas consist of macrophages and epithelioid histiocytes and lymphocytes [58]. There are three histological stages: edema, granulomatous infiltration, and fibrosis but the latest is the most severe, and its presence is an independent predictor of mortality [59]. Cardiac granulomas are usually found in the basal septum, AV node, bundle of His, the ventricular free walls, and papillary muscles [60].

Blood test abnormalities are nonspecific but may reveal raised levels of ACE because it is produced by noncaseating granulomas. ECG findings include conduction abnormalities, ventricular arrhythmias (including ventricular tachycardia), and atypical infarction patterns.

There is a wide spectrum of echocardiographic abnormalities that vary from wall thickening due to edema and infiltration, to wall thinning due to fibrosis. Ventricular chambers may be normal or dilated with regional wall motion abnormalities or globally impaired systolic function. Scar retraction may lead to aneurysm formation, particularly after corticosteroid treatment. Compared to idiopathic dilated and ischemic cardiomyopathy, in CS, normokinetic segments may alternate with affected ones [58] and typically do not show a coronary distribution [61].

**Figure 6:**
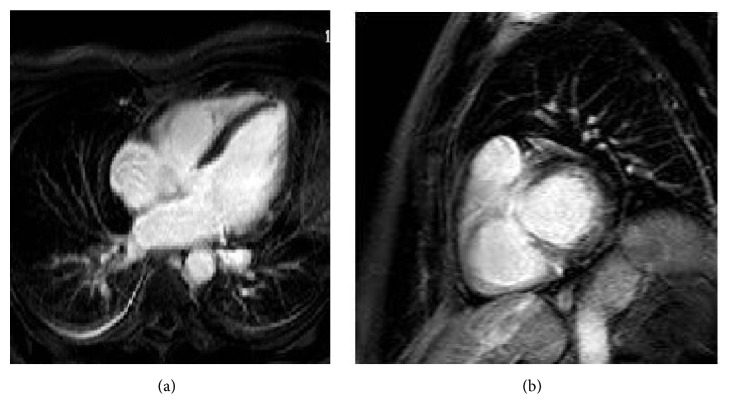
LGE images of a patient with pulmonary sarcoidosis with cardiac involvement. Four-chamber view (a) showing mid-wall focal enhancement in the lateral wall of the left ventricle and in the apex. Note also the enhancement of the mediastinal lymph nodes. Short axis view (b) demonstrating enhancement of the LVOT and possibly of the RVOT. Images courtesy of Professor Dr. Jan Bogaert, University Hospital Leuven.

CMR may display all three stages of edema, inflammation, and scar formation [62]. LGE is patchy and typically found in the myocardium and epicardium of the basal and lateral LV walls [63] (Figure 6), though subendocardial or transmural hyperenhancement has also been observed, resembling an ischemic pattern [35]. The presence, extent, and location of LGE are predictive for the development of ventricular arrhythmias or death and patient response to corticosteroid treatment [59, 64, 65].

Radionuclide imaging with ^18^fluorodeoxyglucose- (FDG-) PET has also been used in the diagnostic work-up [66]. Perfusion imaging is provided with the tracer rubidium and metabolic activity is assessed according to the degree of ^18^FDG uptake. Decreased myocardial perfusion with enhanced ^18^FDG uptake is consistent with inflammation, while decreased perfusion with reduced ^18^FDG uptake is consistent with scarring. As PET identifies active inflammation earlier than CMR [67], hybrid techniques, combining PET and CMR scanners enables simultaneous acquisition of structural and functional data, therefore increasing the diagnostic accuracy [68].

The first international guidelines for the diagnosis of CS [69] incorporated the two modalities, PET and CMR in the diagnostic algorithm. When clinical presentation along with imaging findings set the suspicion, biopsy from an affected extracardiac tissue should be obtained to make the diagnosis probable, with the exception of Lofgren's syndrome (bilateral hilar lymphadenopathy, arthritis, and erythema nodosum with an incidence of 30% of cases) where biopsy is not needed [60].

Definite diagnosis is posed only by EMB but it is not preferred because of its potential risks and low sensitivity (20–30%) [20] due to the patchy distribution of granulomas. PET or CMR directed cardiac biopsy has an increased sensitivity, but still a negative EMB cannot definitely exclude CS.

Treatment of CS incorporates the guidelines directed medical therapy (GDMT) of heart failure with the addition of corticosteroids to reduce the inflammatory process (except asymptomatic hilar adenopathy where no therapy is required). FDG-PET is suggested in the treatment algorithm for the follow-up [69], defining whether satisfactory response to steroids is achieved or a second-line agent (i.e., methotrexate) should be added. If sustained VT or VF occurs, particularly in grounds of myocardial scarring, implantable cardiac defibrillator (ICD) is indicated.

### 5.3. Hemochromatosis

Hemochromatosis is a group of disorders which leads to excessive accumulation of iron within the cells of liver, pancreas, heart, and several endocrine glands leading to cirrhosis, diabetes, heart failure, skin pigmentation, and so forth. It may be hereditary or secondary. In the former, properly called hemochromatosis [70], transferrin iron-binding capacity (TIBC) falls short of plasma iron content while erythropoiesis is normal. Secondary, properly called hemosiderosis, is due to increased catabolism of erythrocytes and occurs in patients undergoing frequent blood transfusions (e.g., in thalassemia major). Cardiac involvement develops later in comparison to other organs and defines prognosis. In the early stages, ECG is normal while with advanced disease low voltage QRS complexes with nonspecific ST-T segment abnormalities and supraventricular arrhythmias. Echocardiographic features may be those of dilated or less often RCM and are not specific [71]. Liver biopsy is the gold standard to establish diagnosis in hemochromatosis. Serum ferritin concentration which has been traditionally used for establishing diagnosis is a rough estimation of total body iron load.

Recently CMR with the use of T2 star (T2^*∗*^) technique has been used to quantify myocardial iron overload [72]. T2^*∗*^ values smaller than 20 msec are predictive of adverse cardiac events [73]. It can also accurately predict the development of heart failure and arrhythmias in patients undergoing repeated transfusions [67]. Some authors advocate its use for the assessment of excess cardiac iron from the early age of 5 years in patients with thalassemia major and suboptimal chelation therapy [74]. Consequently it is regarded the gold standard for follow-up of iron chelation therapy and therapeutic guidance [75, 76].

Treatment by limitation of transfusions, phlebotomy, and chelation can reverse cardiomyopathy in hemosiderosis but liver and/or heart transplantation may be needed in advanced disease and in patients with hemochromatosis [77].

### 5.4. Eosinophilic Endomyocardial Disease

Eosinophilic infiltration of the heart may be caused by a heterogeneous group of disorders, referred to as hypereosinophilic syndromes (HES) and is an uncommon cause of restrictive cardiomyopathy. HES are either primary, first described by Löffler [78], or secondary (Table 3). Eosinophilia is defined as an eosinophilic count greater than 500 eos/mm^3^, while the heart is most commonly affected with a count greater than 5000 eos/mm^3^ [79]. Independent of the underlying causative factor, eosinophilic degranulation causes endocardial damage (Löffler endocarditis) [80]. Primary HES is defined as an eosinophilic count greater than 1500 eos/mm^3^ for more than 6 months in the absence of a secondary cause without other organ involvement [81, 82].

Cardiac infiltration consists of three stages: (a) acute necrotic stage, (b) thrombotic stage, and (c) fibroting stage. The first stage is characterized by inflammation where eosinophilic degranulation releases toxic proteins that induce endocardial necrosis and apoptosis. Rarely, acute necrotizing myocarditis may ensue with high early mortality without appropriate treatment. If left untreated, the first stage evolves into the thrombotic stage due to continuous eosinophilic activation. It consists of thrombus formation over the affected endocardium. Typically there is thrombotic obliteration of left and/or right ventricular apex which may extend to the ventricular outflow tracts, the basal ventricular segments, or even the atria. Thromboembolic complications dominate the clinical picture at this stage. The final stage is due to scarring and irreversible fibrosis of the endocardium (endomyocardial fibrosis, EMF [1]) which may affect the subvalvular apparatus of both atrioventricular valves. Patients at this stage present with advanced heart failure and prognosis is poor.

ECG findings are nonspecific and include sinus tachycardia with conduction disturbances or nonspecific ST-T segment abnormalities. At the first stage, echocardiography may reveal increased LV wall thickness due to interstitial edema, impaired systolic function with abnormal wall kinetics, although most often ventricular systolic function remains preserved. CMR with LGE may additionally reveal endomyocardial involvement, often with patchy distribution [83]. At the second stage, echocardiography shows the presence and location of thrombi while CMR may be more sensitive for thrombus detection [84] and localization. At the third stage, Doppler echocardiography may show echo-dense regions suggestive of myocardial scarring with restrictive filling pattern. CMR, again, is more sensitive and specific for the detection and localization of fibrosis.

The goals of initial therapy are twofold: first to decrease the eosinophil count and attenuate the inflammatory process and second to treat the underlying cause (if any). Corticosteroids are the mainstay of treatment combined with disease specific interventions, for example, antihelminthic therapy for parasitic infections. Anticoagulation should be initiated at the second stage to prevent thromboembolic complications. Once severe EMF has developed, surgical resection with extended endocardial dissection is the treatment of choice but with poor long-term prognosis. Atrioventricular valve replacement should be undertaken at the time of operation if the valves are affected.

### 5.5. Anderson-Fabry Disease

**Figure 7:**
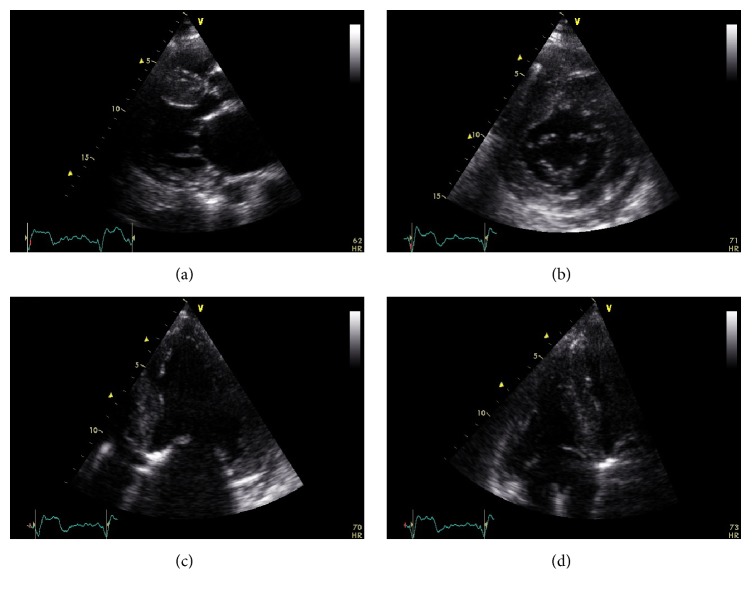
Echocardiographic images from a patient with advanced Anderson-Fabry disease and end-stage renal failure. Parasternal long axis (a), short axis (b), and apical four-chamber view (c). There is left ventricular hypertrophy, more pronounced at the interventricular septum. There is also thickening of the right ventricular free wall apparent at the modified apical four-chamber view (d).

Anderson-Fabry disease is a rare X-linked recessive lysosomal storage disorder, caused by mutations of the GLA gene that encodes *α*-galactosidase A which breaks down neutral glycosphingolipids [85]. This results in intracellular accumulation of glycosphingolipids causing cardiac, renal, and cerebrovascular disease. Males are more commonly affected and diagnosed early in childhood (boys at an average age of 5-6 years and girls approximately 3-4 years older) [86–88] while cardiac involvement occurs in adulthood with a median survival of 50 years. Chronic neuropathic pain, typically affecting hands and feet, can be the first symptom. Children can present with poor growth and difficulties following school activities. Main symptoms are gastrointestinal like postprandial diarrhea and abdominal pain, angiokeratomas, lymphedema, and hypohydrosis. Microalbuminuria, proteinuria, and renal failure present late in the disease process and are a major source of morbidity and mortality [89]. Transient ischemic attacks are also likely to occur. Cardiac disease is caused by accumulation of globotriaosylceramide in all cellular components of the heart. The classic phenotype is that of HCM and less often of the restrictive one. ECG may reveal LV hypertrophy, with preexcitation [90] or prolonged [91] PR interval. Arrhythmias may occur since childhood and include bradycardia, supraventricular tachycardia, atrial fibrillation, or atrial flutter. Echocardiographic concentric LV hypertrophy is the most frequent finding with asymmetric septal hypertrophy developing in approximately 5% of patients [92] (Figures 7(a), 7(b), and 7(c)). RV hypertrophy is a common finding (Figure 7(d)). Systolic anterior motion (SAM) of the anterior mitral valve leaflet, with obstruction of the LV outflow tract, may also occur. Systolic function is preserved and diastolic function is impaired but restrictive physiology is rare [93]. CMR reveals a LGE pattern of the basal inferolateral LV segment and typically spares the subendocardium [35]. Diagnosis in males can be set by measuring *α*-galactosidase A activity or by GLA gene sequencing, while female carriers have a normal enzyme activity and gene sequencing is needed [86].

Treatment includes GDMT of heart failure (with careful beta blocker use) and enzyme replacement therapy which is effective in myocardial remodeling and improves peak systolic strain and strain rate [94] but its use is limited in advanced cases [95]. Patients should receive genetic counseling. Offsprings of affected families may be screened by means of *α*-galactosidase A activity or by GLA gene sequencing for males and gene sequencing for females. This can aid in early diagnosis with early implementation of enzyme replacement therapy before overt end-organ damage occurs.

### 5.6. Diabetic Cardiomyopathy

Diabetic cardiomyopathy (DMCMP) was firstly described as a dilated phenotype with reduced ejection fraction (HFrEF). Recent studies [96, 97] showed a restrictive phenotype as well with preserved systolic function (HFpEF). These distinct patterns have common pathophysiologic mechanisms (autoimmunity, coronary microvascular rarefaction, hyperglycaemia, lipotoxicity, etc.) [98] which seem to be of variable relevance. This distinction is of therapeutic importance as the standard heart failure treatment is of uncertain value for the restrictive DMCMP. Diagnosis of DMCMP with restrictive phenotype (HFpEF) requires exclusion of CAD, valvular, congenital, or hypertensive heart disease, as well as exclusion of infiltrative cardiomyopathies which may require endomyocardial biopsy.

### 5.7. Idiopathic Restrictive Cardiomyopathy

This is the primary restrictive cardiomyopathy, more commonly affecting women, where hemodynamic abnormalities occur without any specific histological changes and in the absence of ischemic, valvular, and congenital heart disease or hypertension. Patients usually present with overt heart failure. Echocardiographic findings are nonspecific as well, with marked dilation of the atria and normal-sized ventricles with preserved systolic function. Doppler demonstrates elevated LV filling pressures [99] more often with restrictive filling pattern.

### 5.8. Progressive Systemic Sclerosis (Scleroderma)

Systemic sclerosis is an autoimmune disorder that practically affects almost any organ system. Myocardial fibrosis results in heart failure symptoms, while fibrosis of the conduction system may lead to ventricular arrhythmias and sudden cardiac death. Physical examination depends on the organs involved with cardiac manifestations mainly caused by right heart failure (peripheral bilateral edema and raised jugular venous pressure). Echocardiographic findings are consistent with restrictive physiology.

### 5.9. Postradiotherapy Cardiomyopathy

Radiotherapy used against thoracic malignancies (Hodgkin's lymphoma, breast cancer, etc.) may provoke several cardiovascular disorders such as coronary artery disease, myocardial fibrosis, valvular heart disease, pericardial disease, and ECG conduction abnormalities. Diffuse interstitial fibrosis after relatively low doses of radiation [100] ultimately leads to decrease in tissue elasticity and distensibility [101] which causes restrictive cardiomyopathy.

### 5.10. Danon Disease

Danon disease is an X-linked disorder, due to primary deficiency of lysosome-associated membrane protein 2 (LAMP2). Symptoms occur in adolescence and include heart failure, mental retardation, and skeletal myopathy. Most patients die of heart failure in the third decade of life [102]. Laboratory tests reveal creatine kinase (CK) elevation two to three times the normal value in males, liver enzyme (aspartate transaminase (AST), alanine aminotransferase (ALT), and lactate dehydrogenase (LDH)) and serum aldolase elevation in at least one-half of patients. As in Anderson-Fabry disease ECG may reveal normal or increased QRS voltage often with extreme hypertrophy (Sokolov score ≥ 50) [103] and/or preexcitation. Other ECG abnormalities include nonspecific intraventricular conduction delay, variable degrees of atrioventricular block, sinus bradycardia, reentrant atrial, and ventricular tachycardia.

Echocardiography reveals severe hypertrophy of both ventricles, resembling HCM but the electrophysiological abnormalities, particularly ventricular preexcitation, may give a clue for differentiating the two conditions [104]. In Danon disease, CMR may show subendocardial LGE [105]. Definite diagnosis is set by biopsy of skeletal muscle. There is no specific treatment.

### 5.11. Friedreich Ataxia

It is an autosomal recessive disorder caused by repeated Guanine-Adenine-Adenine triplets in the frataxin gene on chromosome 9 [106]. Manifestations include neuromuscular impairment of the respiratory muscles with frequent respiratory infections and heart failure. Echocardiography reveals increased wall thickness of the intraventricular septum or posterior wall with preserved systolic function [107].

## 6. Conclusions

Restrictive cardiomyopathies are a group of diseases with various genetic or acquired etiologies. They are characterized by marked increase in ventricular filling pressures which cause symptoms and signs of congestive heart failure. Electrocardiogram, echocardiography, CMR with LGE, or novel radionuclide imaging can direct the diagnosis to specific subtypes of RCM which is important as treatment and prognosis differ significantly, but endomyocardial biopsy is the definite means of establishing the diagnosis. Genetic counseling and familial screening are necessary in hereditary forms.

## Figures and Tables

**Table 1:** Causes of restrictive cardiomyopathy.

(1) Amyloidosis (AL, ATTR, SSA)
(2) Sarcoidosis
(3) Hemochromatosis
(4) Eosinophilic myocardial disease
(5) Idiopathic RCM
(6) Progressive systemic sclerosis (scleroderma)
(7) Postradiation therapy (Hodgkin's lymphoma, breast cancer etc)
(8) Anderson Fabry disease
(9) Danon's disease
(10) Friedreich's ataxia
(11) Diabetic cardiomyopathy (restrictive phenotype)
(12) Drug induced (anthracycline toxicity, methysergide, ergotamine, mercurial agents, etc.)
(13) Mucopolysaccharidoses (Hurler's cardiomyopathy)
(14) Myocardial oxalosis
(15) Wegener's granulomatosis
(16) Metastatic malignancies

**Table 2:** Differential diagnosis between restrictive cardiomyopathy and constrictive pericarditis.

Clinical and investigation features	Restrictive cardiomyopathy	Constrictive pericarditis
History	Systemic disease (e.g., sarcoidosis, hemochromatosis).	Prior history of pericarditis or conditions affecting the pericardium.

Physical examination	± Kussmaul sign, S_3_ and S_4_ gallop, murmurs of mitral and tricuspid regurgitation	Pericardial knock

Chest X-ray	Atrial dilatation	Pericardial calcification

ECG	Low QRS voltages (mainly amyloidosis), conduction disturbances, nonspecific ST abnormalities	Nonspecific ST and T abnormalities, low QRS voltage (<50%)

2D echocardiography	± Wall and valvular thickening, sparkling myocardium	± Pericardial thickening, respiratory ventricular septal shift.

Doppler echocardiography	Decreased variation in mitral and/or tricuspid inflow *E* velocity, increased hepatic vein inspiratory diastolic flow reversal, presence of mitral and tricuspid regurgitation	Increased variation in mitral and/or tricuspid inflow *E* velocity, hepatic vein expiratory diastolic reversal ratio ≥ 0.79 medial *e*′/lateral *e*′ ≥ 0.91 (Annulus Reversus) [4]

Catheterization hemodynamics	LVEDP – RVEDP ≥ 5 mmHg RVSP ≥ 55 mmHg RVEDP/RVSP ≤ 0.33	LVEDP – RVEDP < 5 mmHg RVSP < 55 mmHg RVEDP/RVSP > 0.33 Inspiratory decrease in RAP < 5 mmHg Systolic area index > 1.1 (Ref CP in the modern era) Left ventricular height of rapid filling wave > 7 mmHg

CT	Normal pericardium	Thickened/calcified pericardium

MRI	Measurement of iron overload, various types of LGE (late gadolinium enhancement)	Thickened pericardium

Biopsy	May reveal underlying cause.	Normal myocardium

**Table 3:** Causes of eosinophilia.

Infectious (helminths, HIV, tuberculosis)	Allergic reactions
Inflammatory (Churg-Strauss, Crohn's, Wegener, rheumatoid arthritis)	Drug hypersensitivity (NSAIDS, sulfonamides, antimicrobial)
Malignancies (Hodgkin lymphoma, Non-Hodgkin lymphoma, acute leukemia)	Idiopathic hypereosinophilic syndrome
